# Current role of uniportal video-assisted thoracic surgery for lung cancer treatment

**Published:** 2020-09-02

**Authors:** Luciano Bulgarelli Maqueda, Ricardo A. J. Luengo Falcón, Chiao-Yun Tsai, Alejandro García-Pérez, Anna Minasyan, Diego Gonzalez-Rivas

**Affiliations:** ^1^Department of Thoracic Surgery, Uniportal VATS Training Program, Shanghai Pulmonary Hospital, Tongji University, Shanghai, China; ^2^Department of Cardiothoracic Surgery, Hospital Córdoba, Universidad Nacional de Córdoba, Córdoba, Argentina,; ^3^Department of Thoracic Surgery, Chung Shan Medical University Hospital, Taichung, Taiwan; ^4^Department of Thoracic Surgery, Coruña University Hospital, Coruña, Spain

**Keywords:** uniportal, review, NSCLC, lung cancer, VATS

## Abstract

The use of video-assisted thoracic surgery (VATS) as an approach for early-stage lung cancer treatment has revealed benefits compared to open surgery by minimizing trauma to the patients. This trend has brought the evolution of VATS to less and less invasive methods, eventually leading to the development of Uniportal VATS (UniVATS) technique. This new approach has shown to be resourceful, proving its feasibility even for complex oncological procedures. Furthermore, data is starting to express some benefits over multiport VATS, thus spurring on its development towards newer and more complex procedures. It is also been adopted by the surgical community achieving fast evolution and worldwide diffusion. Here, we review the evolution of UniVATS, its current state of evidence, some basic technical aspects, the present role it has in lung cancer treatment and the ongoing development of the technique.

**Relevance for Patients:**

This article could help patients to understand how the UniVATS technique developed as part of the evolution of VATS, sharing its benefits and indications. Furthermore, patients would be able to understand technical aspects and the current applications of UniVATS for lung cancer treatment.

## 1. Introduction

Since the early 90s, the arising of video-assisted thoracic surgery (VATS) has implied an awakening in thoracic surgery development and interest, particularly for early-stage lung cancer treatment. VATS progressed in the following years grounded on the benefits observed over the open technique [1-5], being simultaneously fed by new emerging technologies, the advance of medical knowledge, new diagnosis and treatment conceptions, and the courage of frontliner surgeons pushing the limits achieving similar oncological outcomes [6] while progressively reducing the trauma to the patient. Following this trend, the technique evolved from the multiport approach to the Uniportal VATS (UniVATS) strategy. The latter has been embraced by the surgical community who is reaching a consensus about its application for early-stage lung cancer treatment [7]. Moreover, its use worldwide expands while the technique shows benefits over previous, more invasive approaches [8-11].

We condense in this review, the evolutionary journey of UniVATS going from its genesis through its progression process, pointing out some basic technical aspects and its current assessment.

## 2. Emergence and Progression of UniVATS

During the past 3 decades, VATS has evolved, showing benefits over the open surgery thoracotomy such as less post-operative pain, better pulmonary function outcomes, and shorter hospital length of stay, all of this implying an enhanced post-operative quality of life [1-5,12]. Once the association between less trauma and better recovery was recognized, the development of VATS technique itself kept progressing in terms of minimizing trauma to the patients, and it was eventually applied as a lung cancer treatment strategy.

Accordingly, the first proposed multiport technique for pulmonary resections (composed of camera port, utility port, and 1 or 2 grasping ports) [13,14] progressed in these terms. For example, the initial size of the ports was reduced over time, as described by Lee *et al*. in the needlescopic technique [15], and later, some groups abandoned the grasping ports adopting a two-port approach [16]. By this time, as part of the biportal technique, the camera had to be placed in the utility port for completion of some steps during the procedure. Hence, eventually, the camera port was left behind, completing the whole surgery through the utility port paving the way for UniVATS surgery for lung cancer treatment.

Addressed first by Migliore *et al*. in 2000 [17], described later by Rocco *et al.*, it was used for low complexity procedures such as spontaneous pneumothorax and lung biopsies [18]. The technique became relevant when it was developed and applied by González-Rivas *et al*. for lung cancer treatment in complex procedures ranging from pulmonary anatomical resections to bronchial and carina reconstructions [19]. Afterward, many groups undertook the UniVATS approach applied to early-stage lung cancer, contributing to its development.

This approach provides a direct view of the targeted tissue and parallel instrumentation, giving the surgical maneuvering a similar feel to that of open surgery instrumentation, setting it apart from other VATS strategies. Maybe this fact is one of the reasons for the growing acceptance and adoption of UniVATS technique by the surgical community worldwide.

An example of this is UniVATS interest groups (UVIG) that have recently emerged [7]. These groups gather thoracic surgeons who prefer UniVATS as a strategy to be applied for early-stage lung cancer patient’s treatment, as well as for other intra-thoracic conditions. These groups, such as the European Society of Thoracic Surgery (ESTS), Uniportal VATS Interest Group (UVIG) or the Japanese Association of Thoracic Surgery (JATS) UVIG, aim to provide a scientific forum that could be the genesis of projects addressing UniVATS to provide qualified data concerning this topic.

Another example of UniVATS acceptance is its implementation in ultra-high-volume centers (UHVC) as the main VATS approach for early-stage lung cancer treatment. To offer context, high-volume centers are defined as those handling from 70 to up to 150 cases per year [20]. In contrast, UHVC perform thousands of these operations annually.

These UHVC are mostly placed in Asia, and the biggest exponent of them is the Tongji University Affiliated Shanghai Pulmonary Hospital (SPH), located at Shanghai, China, which was the seat of a total of more than 17000 thoracic surgeries performed during 2019, being the vast majority of them performed by UniVATS.

Such a high amount of procedures is an ideal substrate for training opportunities. In fact, SPH runs a UniVATS training course and an international fellowship program which have received and trained almost a thousand surgeons from all over the globe. The value of these training programs is evident in light of the recent consensus published by the UVIG of the ESTS, stating that surgeons should be proctored at the beginning of their UniVATS experience, and 50 lobectomy cases are needed to overcome the learning curve [7]. Furthermore, the efficacy results over simulation and virtual-reality-based programs are unclear [21,22]. Then, we can understand why opportunities like the one provided by SPH are valuable postgraduate options to get clinical experience in short periods of time [23].

Based on all we have exposed until now, we can observe that UniVATS has emerged as a result of the evolution of a trend, started by multiport VATS, that implies minimizing trauma to the patient aiming to achieve better post-operative results.

Since then, UniVATS has experienced an interesting and explosive phase of growth with increasing acceptance by the surgical community. Furthermore, we observe an increased number of publications on this matter every year. However, unlike its exponential growth in interest, use, and acceptance, scientific analysis over UniVATS is rising at a slower pace [24].

## 3. Current Evidence on UniVATS

Throughout the evolution of VATS, it has gradually shown benefits over the open thoracotomy [1-5]. In addition to the already mentioned benefits having an impact on a better post-operative course and quality of life (QOL), some data have also expressed that VATS achieved better, or at least comparable, long-term survival rates, and similar oncological results compared to open surgery for early-stage lung cancer treatment [12,25-27]. Furthermore, it has been stated that the mediastinal staging and the resection completeness are equivalent while performed by VATS [6,28]. And beyond this, recent documents explained VATS could have analogous results when contrasted to open thoracotomy for Stage II (cN1) NSCLC [27]. Consequently, there are international guidelines recommending VATS over open technique for early-stage non-small cell lung cancer (NSCLC) [29]. Moreover, analyzing data available to this writing, UniVATS achieved similar or slightly better postoperative course results and lymph nodes assessment when contrasted to the multiport approach for early stage NSCLC treatment [8-11,30-33].

Therefore, it is natural then to infer that the long-term survival and oncological results described for VATS could be riposted also by UniVATS. This could be one of the reasons the surgical community is adopting it even when, to date, we lack stronger evidence about the benefits of UniVATS over multiport, which is currently the established technique [24,34]. An example of this rapid acceptance in spite of current limited research is the data that emerged from the recently established UVIG from the ESTS, reaching consensus on the UniVATS lobectomy. In this paper, they concurred about its definition, indications, contraindications, and perioperative management [7], making clear that the practice has spread widely.

At present, there are enough data to state that UniVATS is feasible and safe to implement for many clinical situations as well as anatomic lung resections in the context of early-stage NSCLC treatment in selected patients [34,35]. In addition, every year much more complex procedures are being performed, providing data about their feasibility, such as the case of locally advanced disease, tracheal or carinal reconstructions, and less invasive UniVATS approaches such as subcostal, subxiphoid, and non-intubated surgeries [35]. Moreover, there is also a trend offering better quality studies showing improved post-operative QOL and recovery with shorter hospital stay compared to multiport [31,32].

However, if we follow the scientific path of the established multiport approach, objective benefits should be sufficiently proven for UniVATS, including post-operative QOL, inflammatory response, treatment efficacy, mid- and long-term survival results.

Considering UniVATS for major resections applied for lung cancer treatment has started only 10 years ago [19], and ever since then, more and better quality studies are being published on this topic every year. Still, we are far from being able to acknowledge UniVATS as a mainstream established technique. Apparently, the surgical community is very enthusiastic about the topic, but just a small part of it is filling the scientific gap needed to define the role of UniVATS while treating NSCLC.

## 4. Technical Aspects of UniVATS

As we mentioned, the surgical technique is in constant evolution. Due to the amount of interest that UniVATS has attracted, it is been affected by feedback from both the surgical community and the industry, thus incorporating new implementation methods, instruments, and technology. In this regard, many factors play a part in the decision of the technique adopted, such as operating room (OR) size; material resources; human resource’s training; surgeon’s comfort; and so on. The interaction between these and other factors have resulted in different setups, and none of them could be labeled the optimum due to the current lack of a study comparing them and their benefits.

However, we describe here the one currently used in the largest UHVC in terms of UniVATS practice in the world to date, the SPH. Due to its characteristics, it seems to be a very efficient approach with a good balance between the variables mentioned.

### 4.1. Position of the patients

The patient is positioned in a lateral decubitus given for the side of the lesion to be operated on. A dense foam roll is placed below the patient’s thorax to achieve ribs’ spreading in the operative hemithorax. A foam pillow is placed between the legs to prevent decubitus lesions. The patient is fixed to the operating table by adjustable stops in each side of it, avoiding any movement during the operation.

The arm in contact with the table remains extended aside, exposed for anesthetic management. The arm in the side of the operating hemithorax is placed in the same direction but on arm support, ensuring a natural and comfortable position, and also accessible for anesthetic management if needed.

In cases of other UniVATS approaches as subcostal or subxiphoid, the patient will be placed in an intermediate 45° lateral decubitus depending on the side of the lesion, with or without the use of a sternum retractor.

### 4.2. Set up for UniVATS

A scrub supporting arch is placed over the head of the patient. This will ensure an aseptic field for the surgery, and it will also provide access to the patient for the anesthesia team.

The scrub nurse and the main instrument table are located at the right side of the operating table toward the feet of the patient. Besides the main instrument table, a secondary instrument table is placed over the operating table and the patient’s feet. This will be used for placing the most used instruments in each procedure to guarantee their rapid access and availability. This table also allows the position of the scrub nurse to remain unmodified; being compatible with whichever procedure could take place.

The surgeon stays in front of the patient in a comfortable position, with a sufficient range of movement. Moreover, the assisting surgeon is positioned at the patient’s back, beside the scrub nurse. It is worth mentioning that there are camera holders and robotic arms that could be used in a mono-surgeon technique [36], disregarding the need for an assistant or leaving the assistant free to help the surgeon.

The screen, camera, and light source are kept in one side of the room, allowing the free flow of the team members in the OR until the patient and the surgical field is set. After this, the equipment is placed at the head of the operating table, leaving enough space for the anesthesia team to work. The height of the screen should be set at eye level for the surgical team, allowing a natural, ergonomic, and comfortable position.

Once connected, the wires will be placed at the back of the patients leaving their front side and incision clear for the surgeon’s work.

**Figure 1:**
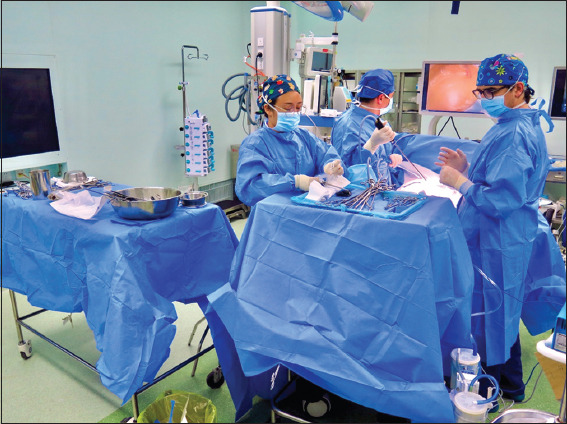
Image displaying the OR set up and team positioning for UniVATS.

Everything remains constant, the only modification for every new case, will be the position of the surgeon and the assistant according to the patient’s position depending on the side of the lesion or the procedure to be performed. This could be a positive factor to achieve efficacy and efficiency in the whole OR team’s functioning (Figure 1).

### 4.3. UniVATS technical tips

Special instruments are required to perform UniVATS with safety. These kinds of instruments are double-jointed with a length between 250 and 400 mm and a shaft width in a range of 5-8 mm, occupying little space in the port. They have different configurations and tip curved angles, and the obvious recommendation would be to have a complete set of instruments. Very important while dealing with complications or to front face complex procedures.

Of course, there are key pieces that could complement a VATS set of instruments to achieve the correct UniVATS performance.

The use of a FullHD camera is important since it allows the better distinction of the different structures to be dissected or transected. The UltraHD cameras are, of course, even more recommendable.

The endoscope used is a 30°, 10 mm thoracoscope. However, thinner thoracoscopes could also be used to achieve more working space in the port.

#### 4.3.1. Incision

In an aseptic condition, after the surgical field is ready, an incision smaller than 4 cm is placed between the middle and anterior axillary line on the 5^th^ intercostal space. Otherwise, for the right upper lobe (RUL) approach, and for segmentectomies in upper lobes, the 4^th^ intercostal space is suggested.

The serratus anterior muscle fibers should be opened without cutting them in a muscle-sparing way. The intercostal muscles should be divided along the upper edge of the inferior rib, trying not to divide a length more than necessary, hence decreasing the trauma to the intercostal space.

After entering the pleural cavity, a wound retractor is placed which opens the wound, prevents blood dripping over the thoracoscope affecting the vision and also prevents wound infections and neoplasm implantation.

#### 4.3.2. Instrumentation

**Figure 2:**
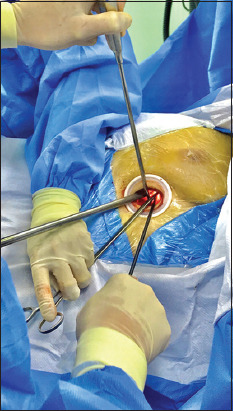
Photography showing the instrument’s position during a subcostal UniVATS procedure.

The UniVATS instrumentation has been compared to a traffic light scheme, where the red light (in the upper or posterior angle of the wound) represents the camera position. The yellow light (the wound’s middle section) is the position for different grasping or suction instruments. And finally, the green light (at the lower or anterior angle) is occupied by the endostapler and other devices. This setting remains constant even during subxiphoid and subcostal approaches (Figure 2).

Following the traffic light scheme, first, the assistant will introduce the camera into the wound, keeping it at the upper position. The surgeon uses ring forceps to position the lung exposing the structures to be dissected. These forceps are usually held by the assistant, while the surgeon uses the curved tip suction and other instruments for the dissection.

While holding the camera, the assistant should always keep the wires toward the backside of the patient, this will provide a clear field for the surgeon’s maneuvers in the front of the patient. The camera should always be kept parallel to the surgeon’s instruments; keeping the same working axis will provide a natural front view and will prevent interfering with the surgeon’s maneuvering.

Due to the set-up and instrumentation, the technique could be demanding for the assistant, especially when addressing inferior pulmonary lobes. This is the reason why the assistant must be well trained in the use of the camera to keep an ergonomic position while giving a proper view, following the surgeon’s work and anticipating the surgical steps. A collaborative attitude is needed to be the “third hand” in case it is needed. A properly trained assistant could save operative time.

Once the surgery is completed, a chest tube is inserted and fixed in the upper part of the wound. If needed, depending on the procedure, a second chest tube could be placed in the lower part of the wound.

## 5. UniVATS for Early-stage Lung Cancer

The advent of lung cancer screening programs in recent time is increasing the diagnosis of earlier stages of NSCLC [37]. The treatment for these lesions is currently mostly addressed by VATS. Wedge resections, segmentectomies, and lobectomies are part of the current armamentarium implemented depending on case and all of them are feasible by UniVATS.

### 5.1. Wedge resection

Small suspicious lesions, that because of their sizes or location, could not have percutaneous histological confirmation during their work-up stage, would be good indications for VATS wedge resections [38]. Consequently, the UniVATS wedge resections have a relevant role in current thoracic surgery, standing as a safe method enabling diagnosis, and in many cases treatment of, solitary infracentimetric peripheral nodules in selected patients [39,40]. The advantage of choosing the UniVATS approach lies in the fact that, if the result of the frozen section practiced to the resected piece required further major pulmonary resection, it could be carried out without changing the approach at all, or if needed, simply by converting to multiport VATS.

A recent study of this technique has exposed the safety of a “tubeless” strategy in which no chest tube is left after surgery when air leaks are ruled out in selected patients [41]. This kind of management could have an impact in the post-operative course and could become an alternative to be chosen for “fast-track” approaches in the context of lung cancer screening programs for the management of small peripheral lesions.

### 5.2. Segmentectomy

For the reasons mentioned before, and the fact that the surgical community faces older patients with later stages of better managed chronic obstructive pulmonary disease (COPD) [42], the sparing parenchima resections, as segmentectomies, have been put in the spotlight.

Segmentectomies are accepted for ground-glass opacity lesions with a diameter <2 cm and a peripheral location in the context of NSCLC suspicion (cT1aN0M0, Stage IA) in patients with limited pulmonary function. There is a trend suggesting the lesions included into the segmental borders, with adequate normal parenchymal margins, followed by lymph node dissection, are related to better oncological outcomes [43].

However, controversy exists as to whether segmentectomy is a viable alternative comparable to lobectomy for the treatment of early-stage NSCLC in selected patients. Therefore, the results of controlled randomized trials currently assessing this issue, as The Cancer and Leukemia Group B 140503 trial (ALLIANCE trial) [44] and the JCOG0802/WJOG4607L study [45], will be welcomed. Both of them finished the enrollment phase and have published perioperative data with comparable results between lobectomy and segmentectomy groups. We expect the final analysis of these studies to add valuable information clarifying the role of these sub-lobar resections in lung cancer treatment.

Beyond this unresolved issue, at the present time, many groups choose the UniVATS method when performing segmentectomies relying on the slightly better, or at least not worse results, UniVATS showed in several studies when contrasted to other minimally invasive techniques [8-11,30-33,46]. The combination of the potential benefits of UniVATS and those of a sparing lung parenchyma resection could have a positive impact while treating the aforementioned borderline sort of selected patients.

The UniVATS segmentectomy technique was reported by Gonzalez-Rivas *et al*. demonstrating its feasibility and safety applied to lung cancer treatment [47]. Later, Xie *et al*. published the largest reported unicenter series of UniVATS Segmentectomies in 2016 [48], adding important data to our previous knowledge. At present, surgeons are performing a large variety of segmentectomies, approaching the different pulmonary segments separately or in combination, and even performing sub-segmentectomies, providing richly detailed technical reports [49-52]. Having said that we need to put an effort toward, first, clarifying the previous mentioned controversy, and then achieving sufficient quality data comparing UniVATS with other minimally invasive strategies applied to segmentectomies.

### 5.3. Lobectomy

In the present time, lobectomy remains the standard treatment for early-stage NSCLC. The aforementioned benefits of VATS over the open surgery technique showed in several studies still need to reach higher levels of evidence. In this regard, analysis like the undergoing VIOLET trial (ISRCTN 13472721) may fill this existing gap.

A similar situation is currently presented by UniVATS lobectomy. A recent consensus of the UVIG from ESTS has detailed that tumors of a size less than 5 cm (T1-T2b), N0 up to N1 disease, can be considered suitable indications for a UniVATS lobectomy approach [7]. Another recent expert consensus about the optimal approach to lobectomy for NSCLC suggests that UniVATS may be associated with less adverse events and post-operative pain [26,53]. Moreover, the first UniVATS lobectomy in a 9-week-old patient has been reported recently [54]. This could reinforce the idea that, similarly to other approaches, the surgical community has embraced UniVATS without providing sufficiently qualified data about its inferred benefits yet. We hope this concerning issue will change in the future. The UniVATS technique has been meticulously described in several previous publications [55].

A proper lymph nodes (LN) assessment during early lung cancer thoracic surgery provides an accurate mediastinal staging that will have an impact on the patient’s further work-up. Its application has been addressed by multiple studies showing VATS Mediastinal Lymph Node Dissection (MLND) as feasible, achieving equivalent, and even slightly better outcomes on this topic compared to thoracotomy [26,28]. Later, the feasibility of a radical MLND through UniVATS was presented by several authors providing data supporting the idea that UniVATS is comparable to traditional VATS for MLND [55-58]; some authors have even shown an increased number of LN dissected contrasted to the multiport VATS approach [33,59]. Consensus is rising toward performing a complete systematic ipsilateral LN dissection in every patient exposed to lobectomies while treating early-stage NSCLC [7]. There are recent detailed descriptions of the techniques for UniVATS MLND that help to achieve proper LN dissections [60].

## 6. UniVATS for Locally Advanced Lung Cancer

Although the role of the minimally invasive approach for central or locally advanced tumors is not defined yet, many groups, especially in Asia, endorse it. They rely on the mentioned reported benefits of VATS over open thoracotomy, stating that a better and faster post-operative course could have an impact in the patient’s adjuvant therapy efficacy with a possible influence on survival rates [61]. Furthermore, mortality rates are comparable to open surgery, as well as the impact in the local control of advanced lung cancer disease [62,63].

As a result, in recent years, procedures such as UniVATS pneumonectomies, sleeve resections, and tracheal or carina reconstructions are being addressed for lung cancer treatment. These procedures have been proven as feasible by Gonzalez-Rivas *et al*. [64-68].

Referred to pneumonectomies, descriptions of the UniVATS technique have been recently published [69]. Apparently, one of the advantages of this strategy applied to these procedures is a more natural view of the hilar structures, similar to that of open surgery, allowing a more comfortable approach and handling, achieving a safe proximal control of pulmonary vessels and airway.

Being a relatively uncommon procedure due to the high selectiveness applied to the patients, the scientific data are scarce. However, it should be noted that patient selection is crucial. Conversions to open surgery, late during the operation, are related to worse outcomes [62].

Recently, a large series of UniVATS sleeve resections cases were published, demonstrating its feasibility and safety for centrally located tumors, otherwise requiring a pneumonectomy [65]. Some authors have compared the UniVATS approach to open surgery, achieving an improved post-operative course [66]. The technique is richly detailed in recent bibliography [68].

Furthermore, UniVATS tracheal and carinal resection or reconstruction has been reported lately [69]. Authors showed the procedures as feasible and recent reports presented even a non-intubated UniVATS approach [70]. Further experiences and more complex and qualified studies will be needed to assess the role of UniVATS for advanced-stage NSCLC treatment.

## 7. Current Assessment on UniVATS for NSCLC Treatment

### 7.1. Subxiphoid and subcostal UniVATS

As described by Kido *et al*. and Zielinski *et al*. [71,72], the approach was firstly implemented for mediastinal pathologies, and then evolved to the subxiphoid UniVATS for lung cancer treatment. The basis of this approach is the avoidance of intercostal nerve injury derived from the incision and instrumentation during surgery. Recent studies showed significantly lower post-operative pain compared to intercostal UniVATS [73].

The technique also allows approaching bilateral lesions in selected patients without modifying the patient’s position.

A subxiphoid UniVATS left upper lobectomy was reported in 2014 [74]. Later, other authors as Lei Jiang *et al*. have addressed the topic for different procedures, even including pneumonectomies [75-77]. Furthermore, it has drawn interest recently, due to the current development of a single port robot that could be compatible with this tactic, the da Vinci SP by Intuitive Surgical (Sunnyvale, California, USA) [78].

Having said this, the technique has some challenges. For example, the instrumentation becomes problematic due to the position of the instruments and their angles of movement. In addition to this, the farther approach, compared to the intercostal, generally requires longer instruments. Then, while approaching the left hemithorax, the instrumentation is done over the beating heart; thus, some interference may occur, possibly triggering an arrhythmic event. Furthermore, the posterior structures are more difficult to reach, meaning that posterior and inferior segmentectomies and moreover some LN station assessments become extremely hard to achieve. Finally, if a complication occurs, an additional intercostal incision or thoracotomy would be required. These are the reasons why it should only be performed in experienced centers for highly selected patients.

Technical aspects have been detailed in previous publications [76]. We hope for scientific evidence to define its indications and benefits over other techniques.

Another approach firstly developed for mediastinal resections is the subcostal strategy that has been recently implemented for UniVATS procedures [79].

As well as subxiphoid UniVATS, its interest relies on avoiding intercostal trauma [FOTO]. But additionally, it could present lesser potential complications compared to subxiphoid when approaching the left hemithorax, because the instrumentation does not interfere with the heart. Another important remark is the better access to posterior structures. The operative technique has been described in previous publications [35]. It is worth mentioning that it is being addressed in combination with the SP robot, as having the potential to allow the execution of every pulmonary resection [78].

### 7.2. Non-intubated UniVATS

In the last 15 years, the anesthetic approach for thoracic surgery has evolved following the tendency of minimizing trauma to the patients. This new method consists in evading the potential risks related to the general anesthesia (GA) [80], together with specific one-lung ventilation (OLV) related events [81,82].

The principle of this technique is based in an iatrogenic pneumothorax that partially collapses the operative lung, leaving space for the surgery’s maneuvers, in a non-intubated spontaneously breathing patient, under different locoregional anesthesia and sedation methods.

This tactic has been proven feasible for pulmonary major resections in selected patients [83,84], and its use has expanded and evolved to reach even complex UniVATS procedures [85]. Current studies are underway to collect evidence to clarify if there are benefits over the OLV under GA or not, but we can foresee it could have a role in future thoracic surgery in diagnosing or treating NSCLC.

### 7.3. The uniportal robot

Following the evolution of VATS, another significant addition to the field of thoracic surgery is the robotic-assisted thoracoscopic surgery (RATS). It has evolved with technological development in more accurate and powerful systems, enhancing the surgeon’s potential, until now, with multiport strategies. Even when to date, RATS has shown it offers similar results to VATS, but with relatively higher costs [86], it is undeniable than it will play a starring role in the future, and currently, we witness the confluence between the paths of both VATS and RATS meeting in the same spot, the single port robot.

**Figure 3:**
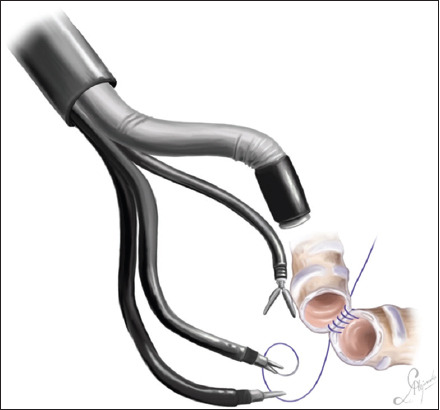
Illustration demonstrating the da Vinci SP performing a sleeve resection during cadaveric tests.

The da Vinci SP system is presently under development. It consists of two components, a free-standing surgeon console and vision cart and a patient-side cart with a single articulated and movable arm that controls up to three wristed instruments and a 3D fully articulated camera (Figure 3).

Similarly to previous da Vinci robotic systems, it concedes motion beyond to what a human hand and wrist could achieve inside the thoracic cavity. It also adds precision to the surgeon thanks to the enhanced view, elimination of physiological tremor, and movement scaling.

A cadaveric test for thoracic surgery procedures by subxiphoid and subcostal approaches has been reported in 2018 [78]. This experience described that this system could overcome limitations observed during subxiphoid and subcostal UniVATS. The instruments achieve a comfortable triangulation of the targeted tissue. All this, together with the aforementioned enhanced view and precision, had a great performance during cadaveric tests performing bronchial anastomosis during sleeve procedures, turning them smooth, fast, and easy.

The characteristics of the system’s articulation provided a better approach to posterior structures, allowing a complete LN dissection. It also allowed a safer approach of the left hemithorax avoiding heart compression. In this way, the limitations observed during subxiphoid or subcostal approaches would be overcome.

However, there still are several disadvantages to be solved in the future. Neither robotic staplers nor suction is incorporated into the system yet. The lack of tactile feedback is a common drawback of robotic systems to date. And last but not least, the costs of this system will face the same argument that its predecessors do.

We excitedly expect the new developments to come in this system, and we also look forward to its first *in vivo* test and clinical setting performance in further studies.

## 8. Conclusion

The current trend in thoracic surgery of minimizing trauma to the patients has fed the development and evolution of UniVATS for lung cancer treatment. The approach seems to be flexible, replicable, and resourceful. Moreover, it could also imply benefits in the post-operative course of lung cancer patients. Despite its explosive growth has brought many new tactics applicable for NSCLC treatment, efforts should be focused on closing the scientific gap in the need for more vast and qualified studies. This would allow to certify if UniVATS implies actual benefits over other minimally invasive techniques applied on the treatment of lung cancer.
